# From detection to remediation: the potential of phosphomelanin in sustainable environmental solutions

**DOI:** 10.3389/fbioe.2025.1724585

**Published:** 2026-01-07

**Authors:** Chang-ye Hui

**Affiliations:** Shenzhen Prevention and Treatment Center for Occupational Diseases, Shenzhen, China

**Keywords:** biosensing, biosynthesis, heavy metals, phosphomelanin, remediation

## Introduction

Heavy metals, such as lead (Pb), cadmium (Cd), mercury (Hg), and arsenic (As), pose significant threats to ecosystems and human health due to their toxicity and tendency to bioaccumulate. These pollutants, often originating from anthropogenic activities, pose a pervasive and persistent global environmental challenge (Hui et al., 2021b; Hui et al., 2023b; Hu et al., 2024; Hui et al., 2024). Current remediation strategies, including chemical precipitation, physical adsorption, and ion exchange, are costly and can introduce secondary pollution (Guo et al., 2021). There is an urgent need for more cost-effective and eco-friendly methods to mitigate toxic heavy metal pollution while minimizing potential risks to human health during the remediation process.

Microorganisms play a crucial role in environmental protection by degrading pollutants and remediating contaminated sites, thereby contributing to the sustainability and resilience of ecosystems (Priyadarshanee et al., 2022). Microbial biotechnology, an effective and eco-friendly solution, has been widely applied to treat heavy metal and metallized waste (Saeed et al., 2022). Microbes can resist heavy metals through their growth and metabolic processes, such as immobilizing and adsorbing them. Notably, melanogenic microbes synthesize melanin and demonstrate superior metal affinity and resistance due to their inherent metal tolerance mechanisms and melanin production, making them advantageous for heavy metal bioremediation (Cordero et al., 2017).

## Innovation in phosphomelanin biosynthesis for heavy metal resistance

A recent study introduced an engineered biosynthesis pathway for phosphomelanin, a synthetically designed melanin variant enriched with phosphate diester bonds that has not been previously reported in natural organisms (Ren et al., 2025), offering a new strategy for heavy metal resistance. The team employed *Bacillus megaterium* and *Escherichia coli* as chassis organisms, leveraging genetic engineering to overexpress tyrosinase (TYR). This enzyme catalyzes the conversion of an exogenously added phosphorylated tripeptide substrate, pSer-Tyr-Gly (pSYG), into phosphomelanin through a multi-stage enzymatic process. The process includes phenol oxidation to quinone (Stage I), phosphate diester bond formation (Stage II), and chain extension (Stage III). Scanning electron microscopy revealed that co-cultivation with pSYG resulted in a roughened bacterial surface, indicating the deposition of phosphomelanin. This melanin not only forms a “biofilm” on the cell surface to adsorb and precipitate heavy metals but also exists in a free state in the culture medium, where its functional groups, such as phenolic, carboxyl, and phosphate groups—commonly reported as key binding moieties in microbial heavy metal biosorbents (e.g., extracellular polymeric substances of *Bacillus* spp. and cell wall components of fungi) —facilitate heavy metal adsorption and precipitation (Nowak et al., 2019; Maumela and Serepa-Dlamimi, 2025).

Compared to other melanins, such as eumelanin and pheomelanin, phosphomelanin exhibits superior heavy metal adsorption capabilities. The study highlights the unique chemical structure of phosphomelanin, enriched with phosphate groups, which significantly enhances its binding affinity for heavy metals, making it more effective in adsorbing and precipitating these toxic ions. This innovation lies in the engineered biosynthesis pathway, which enhances the bacteria’s resistance to heavy metals and provides a versatile mechanism for removing various heavy metals. These findings underscore the significant potential of phosphomelanin for the sustainable and efficient remediation of heavy metal pollution.

## Heavy metal resistance regulation and innovations in visual biosensing

Heavy metal resistance is tightly regulated by specific transcriptional regulators that activate resistance operons in response to toxic metal ions. These operons are designed to be highly specific and sensitive to their target ions, ensuring that resistance mechanisms are activated only in the presence of the corresponding metal ions (Jung and Lee, 2019). This specificity minimizes unnecessary metabolic burden and maximizes protection against heavy metal toxicity. The sensitivity of these operons enables the detection and response to even low concentrations of heavy metals, including those at the nanomolar level, which is crucial for effective resistance mechanisms (Liu et al., 2024; Guo et al., 2025b; Guo et al., 2025c; Liu et al., 2025).

Building on this regulatory framework, our research group has developed a series of whole-cell biosensors by engineering bacteria to produce a series of colorful pigments such as violacein and its three derivatives biosynthesized by the *vioABCDE* gene cluster (Hui et al., 2022b; Hui et al., 2023a; Zhu et al., 2023; Ma et al., 2024), indigoidine biosynthesized by the *bpsA*-*pcpS* gene cluster (Hui et al., 2021a; Hui C.-Y. et al., 2022), anthocyanin biosynthesized by a biocistronic 3 GT-ANS expression cassette (Guo et al., 2022), indigo biosynthesized by four self-sufficient indigo-forming enzymes (Guo et al., 2024a), and carotenoids biosynthesized by the *crtEBIY* gene cluster (Hui et al., 2022c), which change color in response to heavy metals. These pigment-based strategies align with broader efforts by independent teams, including the development of engineered living materials with multiplexed pigment biosensors (Usai et al., 2023), dynamic metabolite control for screening zinc deficiency (McNerney et al., 2019), and precise control of lycopene for screening macroelements (McNerney and Styczynski, 2017), collectively demonstrating the versatility of colorimetric platforms across diverse chemical and biological frameworks. These pigments serve as visual indicators of heavy metal presence, offering a straightforward and sensitive detection method.

Recently, we extended this approach to mercury detection by leveraging the Hg(II)-responsive MerR regulator to drive the biosynthesis of pyomelanin, a brown pigment (Tang et al., 2025). We harnessed three 4-hydroxyphenylpyruvate dioxygenase (HppD) homologs as reporters. These enzymes catalyze the conversion of tyrosine to pyomelanin, a process explicitly induced by Hg(II). Pyomelanin, a type of melanin, is an oxidized, multi-component product that shares structural similarities with other melanins (Wang et al., 2015). It contains functional groups such as phenolic and carboxyl, which are involved in binding heavy metals (Perez-Cuesta et al., 2020). While pyomelanin may also contain groups similar to those in phosphomelanin that facilitate heavy metal binding, further research is needed to fully elucidate these properties. The production of pyomelanin is directly proportional to Hg(II) concentration, enabling quantitative detection (Tang et al., 2025). This innovation enhances the sensitivity and selectivity of Hg(II) detection, providing a visually intuitive readout and contributing to the development of advanced environmental monitoring technologies.

Hypothesis 1 phosphomelanin biosynthesis for heavy metal detection and remediation

**FIGURE 1:**
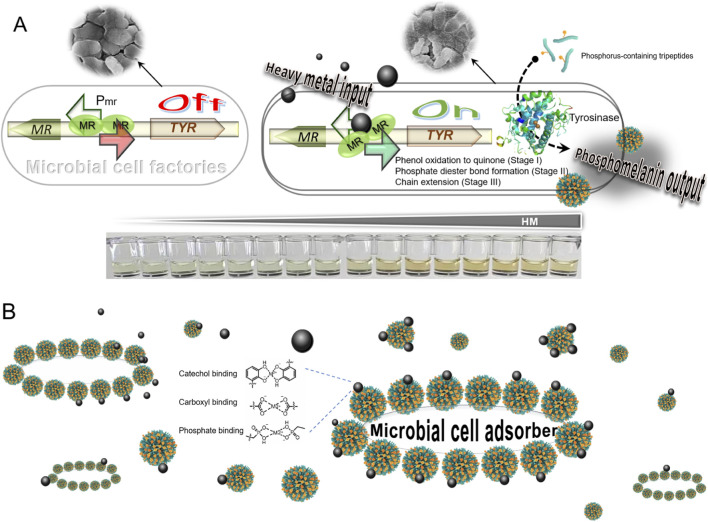
Hypothesis for heavy metal-responsive phosphomelanin biosynthesis and its dual functions. **(A)** Schematic of the genetic circuit. Metalloregulators activate TYR expression in response to heavy metals, producing phosphomelanin via a multi-stage enzymatic process. The resulting phosphomelanin in the culture medium is a colorimetric indicator for heavy metal detection. **(B)** Dual functions of phosphomelanin. It forms a protective “biofilm” on the bacterial surface and exists in a free environment, thereby contributing to the adsorption and precipitation of heavy metals. MR: metalloregulator; Pmr: metal-responsive promoter.

As shown in Figure 1, we propose a novel strategy for heavy metal detection and remediation by integrating the innovative phosphomelanin biosynthesis pathway with our expertise in pigment-based biosensing. We hypothesize that by placing the gene for TYR downstream of metal-responsive promoters recognized by various metalloregulators, we can engineer bacteria to produce phosphomelanin in response to specific heavy metals. This approach leverages the unique properties of phosphomelanin, which provides a visual indicator for heavy metal detection and enhances the bacteria’s ability to adsorb and precipitate these metals. The engineered system aims to combine the specificity and sensitivity of metalloregulators (Jung and Lee, 2019) with the robust adsorption capabilities of phosphomelanin (Ren et al., 2025), offering a dual-function platform for detecting and remediating heavy metal pollution.

The study on the engineered microbial platform for heavy metal resistance via phosphomelanin biosynthesis evaluated the binding capabilities of phosphomelanin for various heavy metal ions, including Cr(II), Pb(II), Yb(III), Dy(III), and Tb(III) (Ren et al., 2025). These metal ions were selected due to their widespread presence in the environment, significant toxicity, and potential for bioaccumulation. Their diverse chemical properties and oxidation states make them representative for assessing the broad applicability of phosphomelanin in practical scenarios. However, this also suggests that phosphomelanin may not exhibit strong selectivity for heavy metals, which presents an opportunity for further development.

## Implementation challenges: biosafety, regulatory considerations, and technical bottlenecks

Based on the insights from the study on heavy metal ion-responsive transcription regulators, we can use various metalloregulators and their known promoters to create sensors and adsorbents for a wide range of heavy metals (Jung and Lee, 2019). For example, we can develop systems responsive to Hg(II) using MerR (Guo and Hui, 2025; Ou et al., 2025), Pb(II) with PbrR (Guo et al., 2024b), Cd(II) utilizing CadR (Hu et al., 2024; Guo et al., 2025a; Hui, 2025), and As(III) through ArsR (Hui et al., 2024). This approach could enable the creation of biosensors that indicate exposure to multiple heavy metals and facilitate broad-spectrum bioremediation. Additionally, it is essential to consider the behavior of phosphomelanin with non-toxic metal ions, such as Ca(II) and Mg(II), which are essential nutrients in biological systems. Given their relatively stable chemical nature, the complexes formed between these ions and phosphomelanin may not precipitate as readily as those with heavy metals, potentially resulting in weaker binding capabilities. This hypothesis requires experimental validation.

The current reliance on exogenously added phosphorylated tripeptide substrates, such as pSYG, for phosphomelanin biosynthesis represents a significant economic and logistical bottleneck for field deployment. The cost and stability of these synthetic peptides likely render large-scale applications impractical. Future efforts must focus on engineering *de novo* biosynthetic pathways within the host organism to produce phosphorylated precursors endogenously. Moreover, the promiscuous metal-binding capacity of phosphomelanin, while advantageous for broad-spectrum remediation, raises critical questions about selectivity and potential interference from competing ions (Ren et al., 2025). It highlights the need to design next-generation tunable pigment-reporting systems that can discriminate between target toxic metals and essential nutrient ions, potentially through directed evolution of the peptide substrate or incorporation of metal-specific chelating domains.

Beyond technical hurdles, real-world application demands rigorous consideration of biosafety and regulatory frameworks. The use of genetically modified microorganisms for environmental remediation is subject to stringent oversight by official agencies to prevent ecological disruption and horizontal gene transfer (Huang and Bai, 2025). We explicitly envision contained deployment scenarios, such as immobilized cell bioreactors, to mitigate the risks of environmental release. Engineering safeguards, including auxotrophic dependencies or kill-switch circuits (Xue et al., 2022), should be integrated into chassis strains. Furthermore, the regulatory pathway for novel biomaterials, such as phosphomelanin, remains uncertain. Toxicity testing, biodegradability assessment, and long-term environmental fate studies will be prerequisites for approval. Addressing these intertwined technical and governance challenges is essential to translate phosphomelanin’s promise from laboratory proof-of-concept to validated, sustainable environmental solutions.
